# Deciphering the Counterintuitive Role of Vascular Endothelial Growth Factor Signaling Pathways in Pulmonary Arterial Hypertension

**DOI:** 10.3390/ijms27020687

**Published:** 2026-01-09

**Authors:** Riccardo Scagliola

**Affiliations:** Cardiology Division, Cardinal G. Massaia Hospital, Corso Dante Alighieri, 202, 14100 Asti, Italy; risca88@live.it; Tel.: +39-340-732-6833; Fax: +39-0141-486-006

**Keywords:** pulmonary arterial hypertension, angiogenesis, vascular endothelial growth factor, SU5416, lung angio-obliteration

## Abstract

Vascular remodeling and progressive lung vessel obliteration are a histopathological cornerstone for the onset of pulmonary arterial hypertension (PAH). However, the role of vascular endothelial growth factor (VEGF) signaling pathways in the development of histopathological vascular changes in PAH is still incompletely understood. This educational review aims to untangle the opposing and heterogeneous actions of VEGF and the receptors it engages in triggering lung angio-proliferative lesions, driving hemodynamic changes in PAH. A proposed ‘VEGF-oriented’ approach attempts to untangle some of the contrasting and complementary actions of VEGF in the pathogenesis of the disease. Experimental models provide a cogent explanation for dysfunctional angiogenesis and the paradox of VEGF-receptor-blockade-induced PAH. The multifaced properties of VEGF, whether angiogenic or nonangiogenic, vary depending on the nature of the ligand, receptor-dependent and -independent signaling pathways, and the duration of the ligand–receptor engagement. Further investigation is needed to translate the knowledge acquired to human subjects and to confirm the pathogenic mechanisms surrounding the phenotypic shift to apoptosis-resistant, hyperproliferative cellular subset and the development of angio-obliterative lesions in PAH.

## 1. Introduction

**Figure 1:**
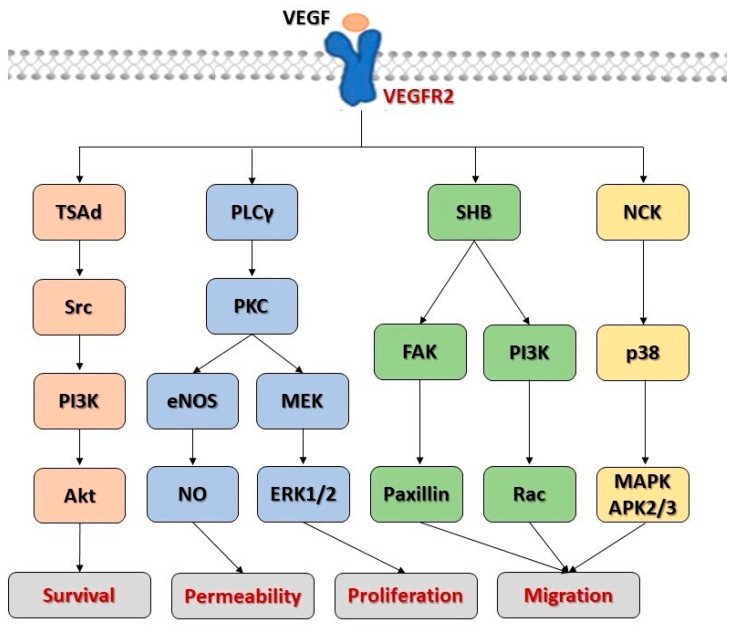
Schematic view of VEGF/VEGFR2 signaling pathways during angiogenesis. Abbreviations: Akt: protein kinase B; eNOS: endothelial nitric oxide synthase; ERK1/2: extracellular signal-regulated kinase 1 and 2; FAK: focal adhesion kinase; MAPKAPK2/3: MAPK-activated protein kinase 2 and 3; MEK: mitogen-activated protein kinase or extracellular signal-regulated kinase; NCK: non-catalytic region of tyrosine kinase; NO: nitric oxide; PI3K: phosphatidylinositol 3-kinase; PLCγ: phospholipase Cγ; PKC: protein kinase C; Rac: actin cytoskeletal regulators; SHB: SH2 domain containing adaptor protein B; TSAd: threonyl carbamoyl adenine synthase.

Despite current advances in therapeutic approaches, pulmonary arterial hypertension (PAH) remains a debilitating disease with a high morbidity and low survival rates in the general population. Therefore, research efforts are focusing on redefining and clarifying the molecular mechanisms of the disease as new promising therapeutic targets [1]. In this regard, dysfunctional angiogenesis has long been believed to actively influence the pathogenesis of PAH. While a large number of genes and proteins, mainly related to the bone morphogenetic protein (BMP) signaling complex, have historically been the focus of attention for their pathogenic role in human end experimental PAH, the molecular disease concept surrounding vascular endothelial growth factor (VEGF) and its receptors has not yet received adequate consideration. Specifically, VEGF plays a pivotal role in promoting angiogenesis and vascular homeostasis by modulating endothelial cells survival, proliferation, migration, and vascular permeability [2] (Figure 1). However, its role in the pathobiology of PAH is still incompletely understood [3,4]. This educational review aims to untangle the opposing and heterogeneous actions of VEGF and the receptors it engages, in order to explore the role of VEGF signaling pathways in triggering pulmonary vascular remodeling and lung angio-obliteration in the pathogenesis of PAH.

## 2. Histopathology of Vascular Lesions in PAH

Histopathological changes in PAH encompass a progressive architectural impairment affecting several cell types at different layers of the pulmonary vascular bed. Constrictive lesions include several histopathological changes in the pulmonary vascular wall, leading to an increase in the cross-sectional area of pulmonary vessels without angio-obliteration. This includes intimal and adventitial thickening as well as medial hypertrophy. The latter is characterized by both hypertrophy and hyperplasia of smooth muscle fibers, along with an increase in connective tissue matrix and elastic fibers and the extension of smooth muscle fibers into non-muscularized pulmonary vessels [5]. On the other hand, the angio-obliterative hallmarks of PAH include concentric lesions and plexiform lesions. The first ones include ‘onion-skin-like’ laminar obliterative lesions composed of extracellular matrix, inflammatory cells, proliferating and migrating myofibroblasts, and smooth muscle cells, as well as a tendency of endothelial cells and fibroblast to acquire a smooth muscle phenotype [6,7]. Plexiform lesions are the most representative angio-obliterative lesions of PAH in humans and animal samples. They consist of a focal plexus of endothelial channels lined by myofibroblasts, smooth muscle cells, and connective tissue matrix progressively obstructing and obliterating small pulmonary arteries and arterioles, especially those with an outer diameter ≤ 50 µm. This in turn leads to the expansion and partial destruction of the pulmonary arterial wall, in parallel with the expansion of such histopathological lesions into the perivascular connective matrix [8,9,10]. Another structural change observed in PAH is represented by lung vascular rarefaction or pruning. This process is characterized by the loss of blood vessels (defined as a reduction in the ratio of the number of blood vessels to the number of alveoli in the intra-acinar regions of the lungs) [11]. These architectural vascular changes lead to a progressive increase in mean pulmonary arterial pressure and pulmonary vascular resistance, either because of narrowing and/or obliteration of the pulmonary vascular lumen as well as in a reduction in paralleled vascular pathways through pulmonary circulation [12,13]. This results in increased right heart workload, followed by right ventricular (RV) hypertrophy and decompensation [14].

## 3. A ‘VEGF-Oriented Perspective’ for Unravelling the Pathogenesis of PAH

Further insights have been provided to improve knowledge of signaling pathways driving vascular remodeling and histopathological alterations in PAH. In this scenario, an in-depth analysis of structural lung vascular changes during disease progression and treatment in humans is extremely challenging, as investigation in patient samples is mostly restricted to the final stages of the disease [15,16]. For this reason, animal experimental models have long been used to gain further insights regarding VEGF signaling pathways involved in histopathological changes responsible for PAH development and progression. Nevertheless, most experimental models have been criticized as differing from the pathogenic features of vascular remodeling observed in human PAH phenotype. Specifically, the most frequently used experimental models include the following: (i) a single application of the plant pyrrolizidine alkaloid monocrotaline inducing PAH; (ii) chronic exposure to hypoxia in rats and mice; (iii) a combination of monocrotaline application and left pneumonectomy. However, although PAH experimental models have provided critical insights into the pathophysiological mechanisms of the disease, to date they have not adequately investigated the role of VEGF in contributing to the pathogenesis of PAH and the development of the associated histopathological changes [17,18,19]. Since VEGF is essential for maintaining vascular homeostasis and endothelial cell physiological function and signaling, a ‘VEGF-oriented’ perspective has been proposed for better elucidating the pathogenic mechanisms of PAH by using a new rodent model in which a VEGF receptor kinase antagonist may be instrumental in shedding light on the contribution of VEGF in the development of the disease [20,21].

## 4. VEGF Receptor Antagonisms for Triggering PAH

As VEGF plays a pivotal role in endothelial cell homeostasis and signaling pathways, it is theoretically plausible that inhibition of VEGF function may trigger apoptosis-resistant endothelial cell proliferation, vascular narrowing, and lung angio-obliteration, predisposing for the development of PAH [22,23,24,25]. In this regard, the compound SU5416 (also named Sugen or Semaxanib) has been identified as a powerful inhibitor of the cytoplasmatic segment of the three tyrosine kinase vascular endothelial growth factor receptors (VEGFR): VEGFR1, also named fms-like tyrosine kinase-1 (Flt-1); VEGFR2, also known as fetal liver kinase-1 (Flk-1) or kinase insert domain (KDR); and VEGFR3, also named fms-like tyrosine kinase-4 (Flt-4) [22,26,27]. SU5416 was first discovered through a screening process designed to identify compounds for the potential treatment of solid tumors through angiogenesis suppression. SU5416 is known for its prolonged activity, due to its high lipophilicity, and for inducing significant antiangiogenic and antitumorigenic effects (e.g., lung endothelial cell apoptosis with airspace enlargement and emphysema, loss of small pulmonary vessels, inhibition of corneal neovascularization, reduction in post-ischemic endothelial cell proliferation, and modulation of tumor microenvironment, either by directly targeting cancer cells and modulating the tumor’s surrounding immune responses) [3,28,29]. These above-mentioned actions are due to the systematic suppression of a broad spectrum of receptor tyrosine kinases (RTK), including VEGFR, platelet-derived growth factor receptor-β, FLT-3, c-Kit, c-MET, and RET [30,31]. However, experimental data underscored the pathobiological action of SU5416 when combined with a powerful trigger of PAH—e.g., chronic hypoxia, shear stress, inflammation, and autoimmune mechanisms [17,20,21]. In this context, the effects do not reflect the enhanced antiangiogenic properties but rather appear to be almost unexpected and, at first glance, counterintuitive, raising further concerns about the heterogeneous role of VEGF in the pathogenesis of PAH. In this regard, the spatiotemporal expression of VEGFR1 and VEGFR2 has been shown to provide useful findings for better understanding the pathogenic mechanisms underlying the development of PAH. Data from lung specimens of adult mice underscored a distinct pattern of distribution of VEGFR1 and VEGFR2 in the endothelium of lung vascular tree. Specifically, VEGFR2 has been shown to be selectively expressed in the endothelial cells of small lung vessels (including pulmonary arterioles), while VEGFR1 is broadly expressed in the endothelial cells of large–small-sized lung vascular bed. Additionally, VEGFR2 selective deletion and hypoxia have been shown to synergistically exacerbate lung vascular remodeling and worsen PAH by promoting arteriolar medial thickening and arteriolar neointimal formation. Taken together, these observations highlight the pathogenic relevance of VEGF/VEGFR2 signaling in angiogenesis and vascular homeostasis, compared to the less prominent role of VEGFR1, acting as a decoy receptor which negatively modulates the VEGF activity by preventing the activation of VEGFR2 [32,33].

## 5. VEGF and BMP Signaling Interplay: Its Role in the PAH Gene–Disease Relationship

VEGF and BMP signaling pathways are strictly interconnected by a bidirectional relationship in the pathogenesis of PAH. In this way, a breakthrough in understanding the pathogenic role of BMP signaling impairment in PAH development was the discovery of the heterozygous germline mutations in the gene encoding the type 2 receptor of the BMP signaling pathway (BMPR2), which are responsible for over 70% of heritable PAH cases and near 15–20% of nonheritable PAH cases [34]. These genetic findings strongly support the crucial role of dysregulated BMP signaling in the endothelium in developing PAH. Specifically, in pulmonary endothelium BMPR2 acts as a key mediator of vascular homeostasis by regulating cell survival, proliferation, and migration. Interactions between VEGF and BNP signaling networks are physically provided by VEGFR3. Under physiological conditions, VEGFR3 plays a protective role by favoring BMPR2 endocytosis, which is necessary for a proper downstream signaling. Under pathologic circumstances, dysfunctional BMP signaling (often compromised by BMPR2 gene mutations) weakens its homeostatic role on the pulmonary vasculature, and increased VEGF signaling activity promotes dysregulated angiogenesis in the context of PAH. Additionally, in vitro and in vivo reduction in or loss of BMPR2 expression in lung vessels have been associated with endothelial impairment, triggering mitochondrial dysfunction and promoting a pro-inflammatory and pro-apoptotic state, which in turn induce endothelial cell apoptosis and exacerbate lung vascular permeability [35]. Loss of BMPR2 expression and enhanced TGF-β activity have been also associated with endothelial-to-mesenchymal transition (EndMT) in human and experimental PAH, with a progressive change of endothelial cells into a mesenchymal phenotype, and its involvement in pulmonary vascular remodeling [36,37,38]. Beyond the major involvement of the BMPR2 gene, further genetic variants with definitive evidence for PAH causality have been identified in recent decades [39]. In this regard, Eyries et al. preliminary identified a novel form of heritable PAH linked to a loss-of-function mutation in the KDR gene (encoding for the VEGFR2) in two index cases from two different families after prospectively screening a series of 311 unrelated patients referred for PAH genetic investigation [40]. The Bayesian methodology analysis provided by Swietlik and colleagues, furtherly provided a strong statistical association between such rare KDR genetic mutation and a specific PAH phenotype, characterized by a reduced transfer coefficient for carbon monoxide and older age at PAH diagnosis [41]. Several genetic mutations with validated evidence supporting the PAH gene–disease relationship have been shown to encode proteins belonging to the BMP signaling complex, which are highly expressed in vascular endothelial cells. Further genetic mutations identified in PAH cases involve the growth differentiation factor 2 (GDF2) gene, encoding for the bone morphogenetic protein 9 (BMP9), which plays a pivotal role in preventing apoptosis and enhancing monolayer integrity of endothelial cells in lung vasculature [42]. Finally, up to 10% of PAH cases have been shown to be linked with mutations in activin-like receptor kinase 1, endoglin, SMAD family member 9, and caveolin-1 genes, encoding for ligands involved in colocalization of BNP receptors, and downstream activation of BNP signaling pathway [39,43,44]. These data underline the complex and intricate interplay between VEGF and BNP signaling networks, and the multifaced role of these dysfunctional pathways in the PAH gene–disease relationship.

## 6. SU5416 in PAH Experimental Models

### 6.1. SU5416 and Chronic Hypoxia

Chronic hypoxia is known as one of the most powerful vasoconstrictive triggers in animal samples, with exposure resulting in a vascular remodeling process in most mammalians. For this reason, chronic hypoxic exposure has long been used for deciphering the pathogenic mechanisms of vascular remodeling in PAH [45,46]. However, a standardized experimental rodent model involving chronic hypoxia alone has been shown to be reversible and has long been known to induce histopathological changes largely limited to the vascular media layer, with no angio-obliteration of small pulmonary vessels [3,17]. On the other hand, although this vasoconstrictive injury alone is not sufficient for triggering PAH, the results of a hybrid SU4516/chronic hypoxia rodent model proposed by Taraseviciene-Stewart et al. in 2001 were surprising [47]. Contrary to expectations, the combined effects of VEGFR antagonism by SU5416 and normobaric chronic hypoxia elicited severe PAH, with increased pulmonary arteriolar muscularization, endothelial cell proliferation, neointimal formation, and the development of angio-obliterative lesions in small lung vessels [27,47]. These histopathological alterations led to significant hemodynamic changes, with a progressive increase in pulmonary flow resistance and right-side intracavitary pressure, thus inducing RV failure and poor prognostic outcomes. Additionally, the degree of pulmonary hypertension was linearly related to the number of fully or partially occluded pulmonary arterioles. In light with this, the SU5416/chronic hypoxia rodent model was proved to be particularly robust and reproducible to human PAH, as the disease in rodents was shown to be severe, progressive, and refractory to drug treatment, as in most cases of PAH in human samples [3,48,49,50,51]. In the same way, Winter et al. reproduced the effects of disrupted VEGF signaling in an experimental model of endothelial cell-selective VEGFR2 knockout mouse model of chronic hypoxic PAH. Compared to VEGFR2 knockout mice exposed to a normoxic environment, those under chronic hypoxia showed a significant PH phenotype, with increase in pulmonary arterial wall thickness, enhanced vascular muscularization, and total vessel obliteration by endothelial cell proliferation and EndMT, resembling the histopathologic lesions of PAH in human samples. Interestingly, the same histopathologic vascular lesions were found in lung specimens of subjects receiving anti-VEGF treatment [52]. These observations underscore the mechanistic role of VEGF/VEGFR2 signaling network for the maintenance of pulmonary vascular homeostasis.

### 6.2. SU5416 and Athymic Rodents

Autoimmune disorders and viral infections have long been known to be associated with an increased incidence of PAH. This clinical scenario raises the suspicion that pulmonary inflammation may be directly involved in the pathogenesis of PAH [51,53,54]. In light of this, the potential causative role of inflammation in PAH development has been investigated by combining the effects of SU5416 treatment with the lack of T cells in athymic rats. Although depriving rodents of a T cells source was expected to provide a less inflammatory substrate for triggering PAH, this experimental data surprisingly showed a worsening of PAH in rats deprived of the thymus [3,55,56,57]. Additionally, in this animal specimen the inflammatory pattern was paradoxically worsened and mainly overexpressed by macrophages, B cells and anti-endothelial cell antibodies. These observations were partially explained by investigating the potential role of T_reg_ cell activity in modulating the inflammatory triggers in these sample models. Specifically, the repletion of inbred euthymic regulatory CD4+ T cells, combined with SU5416 treatment blunted the development of PAH, with limited peri-arterial inflammation and endothelial cell apoptosis [53,58,59]. These findings provide further insights into the role of a pathologically compromised T_reg_ activity in the development of PAH by immune dysregulation.

### 6.3. SU5416 and Ovalbumin Immunization

Although lung vascular inflammation is known to actively influence the development of PAH, its contribution in the pathogenesis of the disease remains unclear. Experimental data from mice with ovalbumin-induced allergic inflammation, showed that the ’single hit’ effect of ovalbumin immunization alone showed muscularization of pulmonary arterioles as a consequence of T helper type-2 cell immune response but no angio-obliterative PAH [3,60,61,62]. By contrast, a hybrid model created by combining the effect of VEGFR-blockade-induced endothelial cell apoptosis by SU5416 with ovalbumin immunization has been shown to induce severe angio-obliterative PAH. Additionally, 20% of the rodents died from RV failure within eight weeks of starting SU5415/ovalbumin treatment [50,60]. In this scenario, initial endothelial cell apoptosis triggered by the VEGFR blockade itself played a significant role in modifying the ovalbumin-triggered immune response. In fact, both B and T cells express VEGFR, which can reduce the activity of the innate immune system by inhibiting dendritic cell function [51,53,59]. Therefore, VEGFR1- and VEGFR2-blockade-induced endothelial cell apoptosis by SU5416 provided a pathophysiological substrate for eliciting a T helper type-2 cell adaptive immune response by ovalbumin-triggered inflammation. This has a tendency to increase lung levels of hypoxia-induced factor-1α (HIF-1α), interleukin-6, and interleukin-13, which, in turn, act as mediators of endothelial cell proliferation. Finally, the combined SU5416/ovalbumin model also provided further insights into the role of B cells in the development of PAH and vascular remodeling [63,64,65]. This effect seems to be enhanced by regulatory T cell suppression triggered by VEGFR blockade. As a counter-test, B cell depletion using an anti CD20 antibody (Genentech) has been shown to prevent both pulmonary vascular remodeling and PAH development, suggesting the pivotal role of B cell immunization in PAH development in this SU5416/ovalbumin experimental model [66,67,68].

### 6.4. SU5416 and Transforming Growth Factor-β1 Overexpression

The transforming growth factor-β (TGF-β) superfamily ligands encompasses several cytokines responsible for regulating the transcriptional program of a broad range of cell types forming the lung vascular wall [69]. Experimental data investigating the enhanced activity of the TGF-β pathway and overstimulation of its type I and II serine–threonine kinase receptors underscored several histopathological changes—e.g., lung vascular muscularization, perivascular fibrosis, EndMT, and enhanced extracellular matrix remodeling—without angio-obliterative PAH [50,70,71,72,73]. By contrast, an experimental model of idiopathic pulmonary fibrosis (IPF) in which adenovirus TGF-β1 was combined with a single infection of SU5416, showed an increased lung fibrosis associated with the development of lung vascular obliteration, vascular pruning, and a significant increase in mean pulmonary arterial pressure [74,75]. Additionally, IPF experimental models also provide further information on the potential interplay between VEGF signaling impairment and lung vascular rarefaction. In this way, data from Farkas et al. showed that adenovirus-mediated overexpression of TGF-β1 in a IPF rat model was associated with decreased levels of VEGF and its receptors, endothelial cell apoptosis in both lung micro- and microvasculature, and microvascular pruning with an overall reduction in vascular density in lung fibrotic areas. Conversely, adenoviral delivery of VEGF in IPF rats resulted in reduced endothelial cell apoptosis, attenuated vascular remodeling, and increased microvascular density. These findings underscore a spatiotemporal relationship between enhanced TGF-β activity, VEGF deficiency, loss of the microvasculature, and lung vascular remodeling in areas of fibrotic damage. As a counterproof, the pigment-epithelium-derived factor, a potent antiangiogenic factor highly expressed in fibrotic lungs, has been shown to play a significant role in promoting vascular rarefaction by negatively modulating VEGF-triggered angiogenic effects on lung vascular tree [11,76].

### 6.5. SU5416 and Left Pneumonectomy

Selective pneumonectomy is known to be a powerful vasoactive agent triggering hypoxic lung vasoconstriction. This is mainly due to an abrupt decrease in the pulmonary vascular bed and the development of post-operative pulmonary oedema, which in turn creates a hypoxic substrate in response to a significant ventilation/perfusion mismatch resulting from the shunt-like effect of blood flowing away from the non-ventilated area [27,77,78]. However, data from animal samples emphasize that selective pneumonectomy alone is insufficient to trigger angio-obliterative PAH. In this context, a hybrid bench model of SU5416 combined with left pneumonectomy showed that the reactive endothelial shear forces caused by a relative loss of pulmonary vasculature, combined with higher blood flow to the remaining pulmonary vascular bed resulted in severe PAH, with initial nonspecific histopathological changes—e.g., adventitial thickening, medial hypertrophy and arteriolar muscularization—followed by endothelial cell apoptosis, neointimal proliferation, and lung angio-obliteration [77,79,80]. The latter, in turn, led to impaired RV function, progressive RV failure, and poor prognostic outcomes [81,82,83]. This model has been shown to be very advantageous as it mimics increased pulmonary blood flow, resulting in pulmonary vascular remodeling comparable to severe clinical PAH in human subjects.

## 7. The Angio-Obliterative Paradox: Critical Insights from the Literature

**Figure 2:**
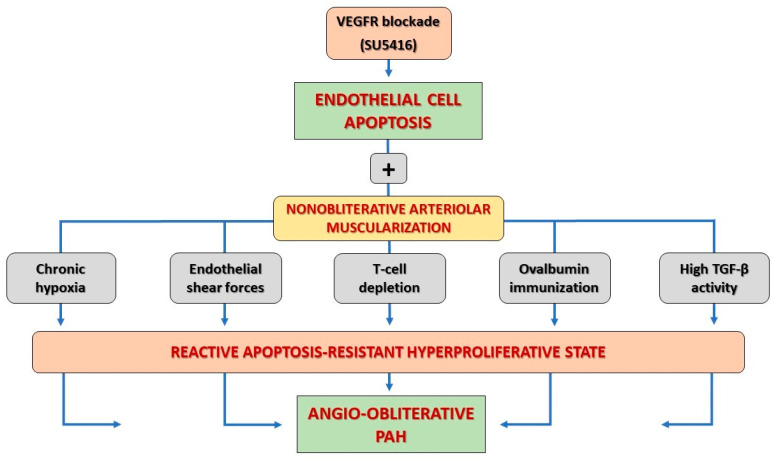
Schematic view of VEGFR blockade-triggered angio-proliferative PAH. Abbreviations: PAH: pulmonary arterial hypertension; VEGFR: vascular endothelial growth factor receptors.

Although the pathogenic role of VEGF signaling pathways in impairing angiogenesis and promoting PAH is still under investigation, the above-mentioned experimental bench models provided critical insights into the pathogenic role of VEGFR blockade in the development of PAH by triggering histopathological structural changes responsible for the obliteration of small pulmonary vessels [20,21,84] (Table 1). While the contribution of the VEGFR inhibitor SU5416 has been shown to be responsible for inducing lung endothelial cell apoptosis by causing chronic inhibition of VEGFR1 and VEGFR2 RTK, it is insufficient alone to trigger angio-obliterative PAH [3]. In the same way, over time it has been shown that chronic hypoxia, immune dysregulation, chronic inflammation, and endothelial shear stress alone only induce nonspecific, non-obliterative lung vascular muscularization, but are insufficient to trigger PAH, as they are unable to induce lung angio-obliteration in the absence of a triggering agent [17,27]. As a result, the ‘double hit’ hypothesis has progressively been accepted as a plausible explanation for lung angio-obliterative lesions deriving from a combined action of acute VEGFR blockade in the presence of another promoting factor (Figure 2). Data from the literature highlight how VEGFR blockade-derived endothelial cell apoptosis plays an essential role as a ‘first hit’ or pathogenic initiator for the development of angio-obliterative PAH lesions by means of a ’second hit’ or promoter [3,40,85]. This evidence is apparently counterintuitive considering the virtual lack of apoptotic cells in plexiform lesions in the prepared lung tissue samples from human subjects with severe PAH. In this pathobiological context, a seemingly discordant response between endothelial cell apoptosis and reactive cell hyperproliferation is instead a common pathogenic mechanism in response to several mechanic, toxic, inflammatory, or immune-mediated triggers [10,15,86]. In this regard, Taraseviciene-Stewart and coworkers reported that increased endothelial cell apoptosis itself in response to the loss of survival signals creates a powerful substrate for the emergence of an apoptosis-resistant endothelial cell phenotype, which in turn triggers PAH by worsening vascular remodeling and inducing hyperproliferative, lung angio-obliterative vascular lesions [47]. By contrast, data from Zhao and Campbell shows that overexpression of endothelial cell growth factors—e.g., VEGF and/or angiopoietin-1—prevented the development of PAH in a monocrotaline-induced rat model [18,87]. Taken together, these findings reflect the paradox that initial endothelial cell apoptosis triggered by VEGFR inhibition promotes the inception of hyperproliferative, apoptosis-resistant cellular phenotypes [3,85].

This counterintuitive concept was elucidated in an experimental model by Golpon and colleagues where endothelial cells—which underwent cellular apoptosis triggered by UV radiation—released growth factors into the growth medium; this fostered the emergence of native endothelial cells resistant to apoptosis [88]. Such a post-apoptotic hyperproliferative state has been further noticed by Sakao et al. in a CELLMAX artificial capillary system, in which human pulmonary microvascular endothelial cells showed an apoptotic response triggered by non-selective VEGFR inhibition with SU5416. When VEGFR-blockade-induced apoptosis was followed by endothelial shear stress, a hyperproliferative state of phenotypically apoptosis-resistant endothelial cells emerged, leading to the development of pulmonary vascular angio-obliteration [89]. In this regard, plausible explanations include the potential release of survival signals by apoptotic cells, which in turn could lead to the selection and proliferation of apoptosis-resistant cellular phenotypes [90,91,92]. In this context, endothelial cell senescence and VEGF signaling are closely entangled in the pathogenesis of PAH. Specifically, senescent endothelial cells are now known to contribute to the exacerbation of PAH by dysregulating VEGF pathways due to increased levels of soluble VEGFR1; this negatively modulates its signaling activity by trapping VEGF and preventing it from communicating with its functional receptors. Conversely, increased VEGF signaling activity is shown to reduce endothelial cell senescence and plays a key role in preserving e lung microvascular density, promoting angiogenesis and vascular homeostasis, and protecting against age-related vascular pruning and impaired angiogenesis [93]. Additionally, the role of endothelial cell senescence in PAH has been furtherly characterized by an initial adaptive response to physiological processes (e.g., endothelial shear stress related to blood flow) or tissue injuries, by secreting endothelial factors like nitric oxide, which, in turn, promote vasodilation and inhibit platelet activation and aggregation. However, in the advanced stages of the disease accumulation of senescent endothelial cells leads to their activation and secretion of vasoconstrictor agents, such as endothelin-1 and pro-inflammatory cytokines, which sustain vascular damage and plays a critical role in the pathogenesis of PAH [94,95]. Alternatively, it could be conceivable that some endothelial apoptotic-resistant precursor cells may be prone to survival and proliferation under a ‘second hit’ stimulus necessary for triggering lung vascular angio-proliferative obliteration and inducing PAH [96,97,98]. Therefore, the proposed ‘Yin and Yang’ approach provides a pragmatic key for reproducing angio-obliterative PAH by means of two synergic and complementary subsets: an initial, acute endothelial cell apoptotic state combined with an additional agent promoting an apoptotic-resistant hyperproliferative state. Furthermore, the emergence of phenotypically apoptotic-resistant endothelial cell hyperproliferation also seems to affect pulmonary arterial smooth muscle cells (PASMC) and trigger the phenotypization and hyperproliferation of apoptosis-resistant PAMSC [89]. This in turn alters their homeostatic balance between apoptosis and proliferation, leading to vessel wall thickening and vascular remodeling [96,97,98,99,100,101].

## 8. VEGF Plasticity on Pulmonary Circulation and Right Ventricular Function

Despite extensive investigation into the pathogenic role of VEGF in the development of PAH, evidence from the literature points out its variable effects during the natural course of the disease [102]. Specifically, at the onset of PAH, VEGF blood concentrations are significantly higher compared to the late stages of the disease. A plausible explanation for this observation is that, in the earliest stage of PAH, VEGF might be synthetized in higher quantities to facilitate pulmonary neovascularization and counteract hypoxic injury [19,22,23]. However, increased VEGF production may also contribute to the development of worsening processes like perivascular oedema and fibrosis, as well as endothelin- and/or leukotrienes-induced pulmonary vasoconstriction [49,83]. Additionally, high VEGF concentrations are locally observed in plexiform lesions, which are known to be histopathological sites for the synthesis of other vasoconstrictors (e.g., endothelin-1 and leukotrienes B4, C4, and D4) [9,10,48]. During the advanced stages of the disease, in parallel with an increase in local VEGF tissue concentrations, the expression of VEGFR progressively decreases, suggesting an impairment of VEGF-related vasodilator properties. Furthermore, as the disease progresses, the impaired homeostatic role of VEGF leads to reduced vascularization, thus contributing to the development of RV failure with a significant impact on prognostic outcomes [101,102,103]. These concerns are supported by murine investigations showing increased VEGF blood concentrations triggered by chronic hypoxia, followed by a progressive decrease in VEGF levels reflecting the development of PAH [104,105]. The relationship between VEGF and right heart function seems at first glance counterintuitive. If the VEGF-related hyperproduction of vasoconstrictive mediators theoretically increases RV afterload, VEGF-triggered vasodilatation through the release of nitric oxide by endothelial cells results in a progressive increase in local blood flow and reduced RV afterload, thereby decreasing intra-ventricular pressure and improving right heart function [101]. The association between VEGF and RV function has been mainly investigated in animal models [18,105,106]. In a monocrotaline murine model, PAH has been shown to develop in VEGF-deficient mice, while VEGF administration reduced RV pressure, suggesting the protective role of VEGF against PAH [105,107]. In line with these data, a negative correlation between RV wall thickness and VEGF plasmatic levels has been observed along with an improvement in RV function, suggesting the molecule’s likely role in blunting the development of right heart failure in subjects with PAH. These observations highlight the critical role of VEGF signaling pathways in RV function and lung vascular homeostasis, and their potential implications in the pathogenesis of PAH at different stages of the disease, with potential consequences on clinical impairment, functional deterioration, and prognostic outcomes [3,101,103].

## 9. ‘Escape Angiogenesis’ and Pathogenic Response to Vascular Triggers

**Figure 3:**
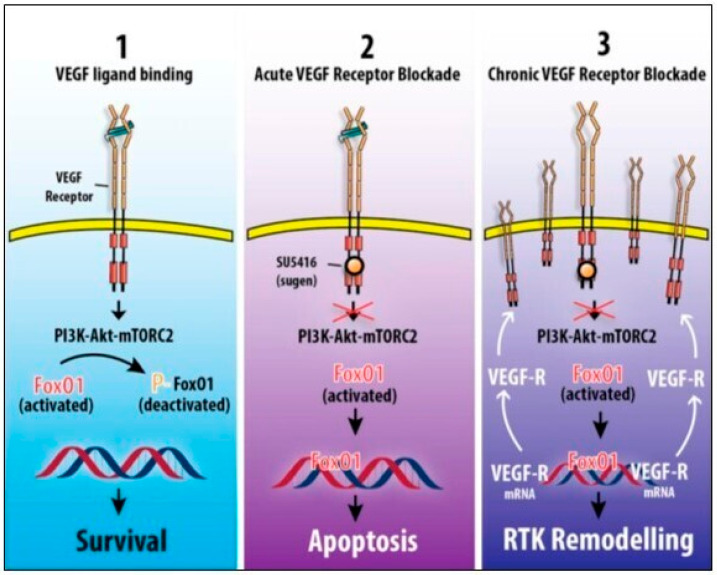
Schematic viewpoint focusing on the central role of the transcription factor forkhead box protein O1 in the control of the following: VEGFR controlled endothelial cell survival (panel 1); VEGFR antagonist-induced endothelial cell death (panel 2); VEGFR2-blockade-induced resistance (panel 3) (Reprinted with permission from Voelkel and Gomez-Arroyo (2014) [3]. Copyright 2014, American Thoracic Society, Inc.). Abbreviations: FoxO1: forkhead box protein O1; RTK: receptor tyrosine kinase; VEGF: vascular endothelial growth factor; VEGF-R: vascular endothelial growth factor receptors.

Our understanding of angiogenesis resistance mechanisms paradoxically triggered by anti-angiogenic drugs (also known as ‘escape angiogenesis’) in human and experimental PAH and their consequences on lung angio-obliteration has substantially improved over the last decades, with several in-depth studies currently underway [3,108]. Experimental data from SU5496 rodent models suggest that angio-obliterative PAH is likely triggered by an initial pharmacological or endogenous apoptotic agent combined with an additional factor promoting an apoptotic-resistant, hyperproliferative process by increasing VEGF circulating levels [50,89]. However, other mechanisms triggering this apoptosis-resistant phenotypic change have been investigated in the context of angio-obliterative PAH. Apoptotic-resistant cellular hyperproliferation can also be triggered by a rebound effect causing high levels of VEGF ligands due to a persistent VEGFR2 blockade [3,109]. Experimental findings from the VEGF-dependent phosphoproteome of endothelial cells incubated with VEGF demonstrate the plasticity of VEGFR signaling pathways, which are associated with the activation as well as deactivation of a broad spectrum of peptides. Specifically, the VEGF-regulated phosphoproteome is linked to the PI3K-mammalian target of rapamycin complex 2 axis; it was identified in the forkhead box protein O1 (FoxO1) transcription factor, which plays a pivotal role in the control of VEGFR2-related endothelial cell apoptosis and survival [108,109,110]. Whereas acute VEGFR2 antagonism blocks phosphorylation and deactivates FoxO1 leading to FoxO1-dependent activation of apoptotic signals, chronic VEGFR2 inhibition leads to the persistent activation of endothelial cell; this triggers RTK remodeling and the development of VEGFR2-blockade-induced apoptosis resistance [109,111,112]. The persistent engagement of VEGF with VEGFR2 causes FoxO1 to be phosphorylated and deactivated, resulting in a decreased expression of cleaved caspase-3, promoting endothelial cell growth induced by VEGF. Conversely, acute VEGFR2 blockade increased FoxO1 expression and induced apoptosis. In cases of persistent VEGFR2 blockade, a FoxO1-dependent feedback loop is responsible for a downstream regulation, which in turn enhances the transcription of VEGFR-mRNA, increases synthesis, and upregulates the expression of VEGFR2. This favors a shift to an apoptotic-resistant hyperproliferative cellular phenotype and leads to angio-obliterative PAH [3,108]. These findings have been confirmed by the observed rebound effect following long-term anti-VEGF therapies with RTK blockers in cancer patients, including a case series of dasatinib-induced PAH, and the increased transcription and overexpression of growth factor receptors genes and proteins, respectively. The observed higher levels of circulating VEGF in patients undergoing long-lasting treatment with the pan-tyrosine kinase inhibitor dasatinib, together with the paradoxical development of reversible PAH in this subset population, point to the RTK remodeling process triggered by chronic VEGF ligand engagement with VEGFR2, which contributes to RTK upregulation and the subsequent hyperproliferative, apoptosis-resistant cellular phenotype, as a liable mechanism of angiogenesis resistance in this pathophysiological context [3,4,50]. Conversely, experimental data have shown clinical and hemodynamic improvements following treatment with the RTK inhibitor imatinib in patients with PAH. These apparently conflicting results take into account different RTK inhibiting profiles targeted by the two VEGFR inhibitors. Specifically, the broad spectrum of RTK inhibition targeted by dasatinib and the blockade of specific RTK like ephrin receptor kinases—which are actively involved in VEGF–VEGFR2 endocytosis and endosomal-dependent degradation—seem to partially explain the pathogenic role of dasatinib in the development of PAH and the divergent therapeutic outcomes compared to other VEGFR antagonists [113,114,115] (Figure 3). Furthermore, the long-lasting triggering of FoxO1-related signaling feedback is also responsible for the enhanced transcription of several receptor-encoding genes engaging other angiogenic factors—including fibroblast growth factor-1R, TGF-βR2, ephrin-B2, epidermal growth factor receptor, and c-Kit—which are actively involved as positive regulators in angiogenic process in PAH [3,116,117]. In the same way, the effects of the multi-target RTK inhibitor SU5416 can be also explicated by alternative mechanisms, not strictly related to the VEGF signaling blockade, like endothelial cell toxicity and the systematic interaction with a broad spectrum of RTK [30,31,118,119,120]. Finally, the main VEGF-related mitogenic, proangiogenic, pro-survival, and permeability-enhancing functions are known to be primarily mediated by the VEGFR2 signaling pathway; however, data from the literature underscored a potential role of VEGFR1—generally acting as decoy RTK negatively regulating VEGF activity by preventing activation of VEGFR2 or sequestering VEGF and decreasing its activity—in transmitting pro-survival signals by triggering the overexpression of the anti-apoptotic protein survivin; this has been associated with the development of plexiform lesions of small pulmonary vessels in PAH [3,22,110]. Taken together, all these pathobiological mechanisms emphasize the versatile role of VEGF and its receptor signaling pathways in modulating angiogenic resistance by favoring a shift to apoptosis-resistant hyperproliferative cellular phenotypes and triggering the development of the histopathological hallmarks of PAH.

## 10. Conclusions

Progressive lung vessel obliteration is a histopathological cornerstone in the development of PAH. In this context, a proposed ‘VEGF-oriented’ perspective attempts to untangle some of the conflicting and complementary actions of VEGF in the pathogenesis of the disease. Experimental models provide a cogent explanation for the paradox of VEGF-receptor-blockade-induced PAH and the multiple properties of VEGF, which can be either angiogenic or nonangiogenic depending on the nature of the ligand, the receptor-dependent and -independent signaling pathways, and the duration of the ligand–receptor engagement. Further investigation is needed to translate the knowledge acquired to human subjects and to confirm the pathogenic mechanisms surrounding the phenotypic shift to apoptosis-resistant, hyperproliferative cellular subsets and the appearance of angio-obliterative lesions in PAH.

## Figures and Tables

**Table 1:** Triggering mechanisms sustaining PAH development in SU5416 experimental models.

SU5416 Experimental Model	Triggering PAH Mechanism
SU5416 and chronic hypoxia	Sustained and widespread vasoconstriction in response to long-standing hypoxic stimulus
SU5416 and athymic rodents	Immune dysregulation by depletion of T_reg_ cell activity and loss in modulating inflammatory response
SU5416 and ovalbumin immunization	Modification of ovalbumin-triggered immune response by enhancing B and T cell activity
SU5416 and TGF-β1 overexpression	Raised lung tissue expression of TGF-β1 and enhancement of its downstream signaling pathway
SU5416 and left pneumonectomy	Increased reactive shear stress related to a relative loss of pulmonary vasculature and higher blood flow to the remaining lung vascular bed

## Data Availability

No new data were created or analyzed in this study. Data sharing is not applicable to this article.
